# Progress in Surgery

**Published:** 1896-08-01

**Authors:** 


### Progress in Surgery.

### SURGERY OF THE PERITONEUM.

**General Septic Peritonitis** is the subject of a paper by Lockwood,[1] who refers to its extreme fatality, although some cases have lately been saved by surgical measures. Success might be greater if an earlier diagnosis could be made. As regards diagnosis, the greatest difficulty is met with in peritonitis due to internal causes. Lockwood[2] thinks: First, that in nearly every case the clinical symptoms of general septic peritonitis are those of acute intestinal obstruction. Second, that in many cases general septic peritonitis cannot be differentiated from acute mechanical obstruction. Third, that to prevent a mechanical obstruction, a gangrenous or ulcerated appendix, a septic Fallopian tube, or a perforation of the alimentary tract being overlooked, an early laparotomy ought to be performed. Fourth, that after operation even the most desperate cases may recover. In peritonitis the most reliable diagnostic symptoms are[3]: (1) The non-passage of flatus or fæces; (2) the abdominal distension; (3) the absence of vermicular movements of the intestine; (4) the collection of fluid in the pelvis; (5) evidences of inflammation above the cæcum, Fallopian tube, gall bladder, or elsewhere; (6) the pulse; (7) the vomiting; (8) the previous history. He lays stress upon the desirability of rapidity and precision in operating, and upon the thorough emptying of the distended intestines, and the prevention of shock and collapse. The kind of peritonitis influences the prognosis. In bacillary peritonitis the effects upon the serous membrane are superficial, and capable of recovery. In streptococcal peritonitis the bacteria penetrate the depths of the peritoneum, and cannot be assailed by ordinary measures. Of ten cases upon which the author has operated three have recovered. Battle[4] reports a case showing the advantages of prompt surgical interference in a case of suppurative peritonitis. A young man, aged 21 years, was admitted three days after he had struck himself over his hip when opening a door. Pain in the abdomen came on soon afterwards, and increased in severity. His bowels had acted, and there had been no vomiting. His temperature was 102·4, the knees were drawn up. the face anxious, and the abdominal walls rigid. There was no distension, but light percussion showed an area of dulness extending from the pubes outwards to about the middle of Poupart's ligament on the right side. A median incision was made above the pubes, and about half a pint of pus evacuated; this occupied the pelvis and right iliac fossa, but was not shut off by adhesions from the general peritoneal cavity. The source of the mischief could not be demonstrated, but was supposed to be in the vermiform appendix. The abdomen was washed out with sterilised water, and a glass drainage tube was put in, the patient making a good recovery. Battle considers that this case shows that it is not desirable to delay operation until the classical signs, including vomiting, have unmistakably proved the presence of peritonitis, for the patient is then exhausted and unable to stand the necessary surgical proceedings.

**Laparotomy for Tuberculous Peritonitis.**—The method of healing in these cases is still a matter of dispute. Jordan's[5] views are as follows: (1) The reason of the cure of tuberculous peritonitis after an operation is still unknown; none of the numerous theories are satisfactory. (2) A complete cure takes place after a laparotomy, and the peritoneum returns to a perfectly normal condition. (3) The *restitutio ad integrum* is the result of the absorption of the tubercular tissue. (4) The formation of adhesions is not necessary in order to bring about a cure. In many cases none are formed. (5) Clinically, the patients may not be cured when they are well from an anatomical point of view. (6) The patients should not be considered cured until some years after the operation. (7) Peritonitis sicca is also curable by a simple abdominal incision. The adhesions become absorbed and the peritoneum returns to a normal state. Israel[6] concludes: (1) That the disappearance of tuberculous peritonitis cannot be explained by the removal of the ascites, for in some of his successful cases there was no ascites. (2) That laparotomy may be followed by recovery even when the peritonitis is combined with intestinal ulceration. (3) That tuberculous tumours of the size of a cherry-stone may disappear within thirty-six days. (4) That hectic fever does not contra-indicate the operation, as some French surgeons suppose. Gatti,[7] as a result of his observations on guinea pigs and rabbits, thinks that healing takes place by degeneration of the epitheliod cells, independently of phagocytosis, without the formation of new connective tissue, contrary to the opinion of many authors. As to the factors which stimulate this process, the author directs attention to the serous fluid, which he found one, three, and five days after operation, and which appears to saturate the tuberculous mass, exerting a bactericidal and attenuating action on the bacilli. Vassilevsky's[8] conclusions on this subject are: (1) The operation alters the histological character of the tuberculous growth in that an increase of the epitheliod cells takes place in them, and diminishes at the same time the number of leucocytes in the tuberculous granulations; (2) the laparotomy checks the general dissemination of the tuberculous process; (3) the operation reduces the number of the bacilli in the tuberculous issue, and promotes the resorption of the products of the decomposition which took place in the tuberculous granulation growth previous to the operation.

Thomson[9] thinks that we must look to the surgical interference *per se* for the true cause of the amelioration of symptoms following laparotomy; the tissues and the bacilli meet on fairly equal terms in the peritoneum, and the operative handling first gives the former the necessary fillip which enables them to gain the mastery. He deduces the following rules for treatment: Medical measures, particularly abdominal massage and copious and regular evacuation of the bowels, are to have a fair trial. These failing, operation is indicated. If free fluid is present, it is to be evacuated, the wound being at once sutured if the fluid is serous, a drainage tube being used for two or three days if purulent. Where the fluid is encysted, operation is also very successful. Where, however, the fluid is slight in amount or absent, and adhesions and matting are prominent features, further knowledge is required as to the indications for operative interference. In such cases operation, though not so favourable as in ascitic cases, often exerts a beneficial influence.

The latest statistics relating to the operation are those of Roersch (quoted by Max Jordan),[10] which present 358 operation cases. Of these 20 (5·59 per cent.) died as a result of the operation; 51 died a few weeks later of other tuberculous trouble; and 250 (70 per cent.) were cured. One hundred and eighteen were kept under observation for at least six months, 79 for a year, and 53 for two years or longer. Mazzoni[11] reports 35 operations for peritoneal tuberculosis, with 33 cures and two deaths, but one death was the result of a resection of the knee for tubercular disease. Rendu,[12] Merklen,[13] and others are strongly in favour of operation.

[1] Brit. Med. Journ., March 21, 1896. [2] Clin. Journ., April 1, 1893, p. 349. [3] Brit. Med. Journ., March 21, 1896. [4] Med. Press, Jan. 1, 1896. [5] Annals of Surg., Jan., 1896, and Beitr. f. Klin. Chir., Bond xii., Heft 3. [6] Lancet, Jan. 18, 1896. [7] Brit. Med. Journ., April 25, 1896, and Gazz. degli Ospedali, April 4, 1896. [8] Brit. Med. Journ., April 25, 1896, and Arch. de l'Inst., Imper. de Med. Exper. St. Petersburg, 1895, Tome iv., No. 3. [9] Brit. Med. Journ., Feb. 8, 1396, and Practitioner, Nov., 1895. [10] Annals of Surg., Jan., 1896. [11] Brit. Med. Journ., Jan. 4, 1896. [12] Med. Week, Dec. 27, 1895. [13] Ibidem.

